# Tetanus Toxin and Antitoxin1Read at the thirty-sixth annual meeting of the American Veterinary Medical Association, September, 1899.

**Published:** 1899-11

**Authors:** Joseph McFarland, E. M. Ranck

**Affiliations:** Philadelphia, Pa.; Philadelphia, Pa.


### TETANUS TOXIN AND ANTITOXIN.[1]

By Joseph McFarland, M.D., and E. M. Ranck, V.M.D.,

PHILADELPHIA, PA.

(Continued from page 646.)

*Discovery of the Antitoxin.* A most important discovery followed the immunization experiments in tetanus. In 1890, Behring and Kitasato[2] found that the blood-serum of animals artificially immunized to tetanus contained a substance which, when the serum was mixed with a virulent culture of the tetanus bacillus, destroyed the virulence of the culture for susceptible animals. This neutralization was entirely independent of the germicidal power of the serum which Buchner had investigated in 1889, for it was subsequently shown that the destruction of the virulence was manifested upon the filtered sterile toxin as well as upon the living bacteria in the culture. It resided in a new substance, an *antitoxin*. The following is a synopsis of the terms which Behring and Kitasato gave their discovery to the scientific world:

1. The blood of rabbits immunized to tetanus possesses the power of destroying the tetanus toxin.

2. This power is demonstrable in the extravascular blood and in the clear serum obtained from it.

3. The peculiarity is so permanent that it remains operative in the bodies of other animals, so that one can, by transfusion of blood or serum, bring about a distinct therapeutic action.

4. The tetanus-toxin-destroying-peculiarity is absent from the blood of animals not immune to tetanus, and if tetanus toxin be introduced into the blood of such an immune animal it can be recognized after death in the blood and body-juices of that animal.

[1] Read at the thirty-sixth annual meeting of the American Veterinary Medical Association, September, 1899.

[2] "Neber das Zustandekomnen der Diphthirric Immunität und der Tetanus; Immunität und der Tetanus; Immunität bei Thirven," Deutsche med. Wochenschr., 1890, No. 49, p. 1113.

In February, 1891, the results of Behring and Kitasato were confirmed by Tizzoni and Cattani.[1] These experimenters found that the serum from an immunized dog when introduced into a normal dog protected it certainly from a fatal dose of the injected culture. They also suggest that as the protective substance in the serum is operative in such small quantities it is probably an enzyme or ferment. Curiously enough, while in their researches Lizzoni and Cattani were able to produce immunity in white mice and dogs by the injection of the immunized serum, they failed to do so in guinea-pigs and rabbits. Continuing their experiments in this field, Tizzoni and Cattani give us[2] information regarding the nature of the protective, or, as it has been called, the "antitoxic" substance. They found that the protection afforded by the serum is lost by elevation of temperature to the point of coagulation of the serum; that the antitoxin is not dialyzable, and that mineral acids—HCE —in the proportion of one-half drop to five drops of the serum entirely destroy it. Their view that the antitoxin is an enzyme is supported by experiments.

Vaillard[3] seems to have been the first to successfully immunize the rabbit. His success depended upon the use of filtered culture heated for one hour to 60° C.

The immunization of large animals was first undertaken by Behring.[4] For the immunization of the horse he used filtered cultures having such toxicity that 0.75 c.c. would kill a rabbit in three or four days. To preserve the toxin he added 0.5 per cent. of carbolic acid. The injections of the toxin were made with the addition of 0.25 per cent. of trichloride of iodine ($ICl_3$) to protect the animal from too intense an action. Beginning with a subcutaneous injection of 10 c.c. of this moderated toxin, the animal received increasing doses similarly given at intervals of about a week until the quantity of trichloride being diminished and finally omitted, and the quantity of toxin increased to a large amount, the blood of the horse is found to contain antitoxin in useful amounts. By this and similar methods of immunizing the animals, Behring was able to produce serums with an immunizing power of 1 : 1,000,000 and higher, the expression 1 : 1,000,000 indicating that 1 c.c. of the antitoxin serum is capable of protecting 1,000,000 grammes of white mouse.

1 Centralblatt f. Bakt. u. Parasitenk., February 16, 1891, vol. ix., No. 6, p. 189.
2 Ibid., May 26, 1891, Bd. ix., No. 21, p. 685.
3 Comt. rend. Soc. de la Biol., 1891, No. 7.
4 Zeitschrift f. Hygiene, 1892, Bd. xii. p. 45.

Brieger and Ehrlich, pursuing somewhat similar methods for the immunization of goats, have succeeded in showing that the antitoxic substance passes from the blood into the milk of the animal, but occurred there in small quantity as compared with the blood.

Brieger, Kitasato and Wasserman (1892) have recommended the use of *thymus bouillon* as a culture-medium for the tetanus bacillus in immunization experiments, the advantage being that in such culture the tetanus bacillus does not form spores and the toxic potency of the bacillus is much reduced. In our judgment this is a very small advantage.

Ehrlich and Tizzoni and Cattani have shown that the offspring of immunized animals are rendered immune to tetanus through the milk of the mother.

In preparing the tetanus antitoxin we have employed the methods suggested by the pioneers in the serum-therapy, modifying as experience suggested.

Much probably depends upon securing an animal with sufficient resisting power. The large experience which our researches upon the diphtheria antitoxin had given us laid important emphasis upon this point, and we have found it best to secure horses that have already recovered from spontaneous tetanus for the purpose. These horses have considerable vital resistance and probably have some antitoxin already present in the blood, though experiments made by us have failed to show it.

The toxin is prepared in the way above described, the bacillus being grown in ordinary bouillon.

Commencing with a dose of 1 c.c. of toxin, of which 0.001 c.c. is fatal to a guinea-pig, the horse receives rapidly increasing subcutaneous injections as often as careful observation of the temperature, muscular condition, etc., will allow, until doses of 300 c.c. or more are given.

Trial bleedings, taken by introducing a hypodermatic needle into the jugular vein and drawing the blood into a sterile syringe, are examined, and when they show the blood to contain a sufficient quantity of antitoxin to be valuable the horse is bled.

The management of the blood and preparation of the serum are in accordance with the often published methods for the preparation of the diphtheria antitoxin. We have found, however, that the tetanus antitoxin is not well preserved by trikresol, and therefore prefer to use phenol in the proportion of 1 per cent. Before leaving the laboratory the serum is filtered through diatomaceous earth, to remove the precipitate made by the phenol.

The antitoxic value of the serum is determined by experiments made either upon white mice or, as we prefer, upon guinea-pigs.

The essential feature depends upon the determination of the exact strength of the toxin—*i. e.*, the least certainly fatal dose, and the estimation of the least quantity of the serum that will protect the guinea-pig against such a dose. As the toxin deteriorates rapidly, losing strength every day after filtration, the tests must always be made with perfectly fresh, newly tested toxin. Our method is to open a bottle of the culture and add phenol one day and filter and test the next day, and to make tests of the antitoxin with new control-tests for the antitoxin as soon as the strength of the toxin is determined. Thus:

1. Tests for toxin:

| | | | 1st day. | 2d day. | 3d day. |
|---|---|---|---|---|---|
| Guinea-pig, | 420 grms. | 0.001 c.c. toxin, | S. | SS. | Dead. |
| " " | 450 " | 0.01 " " | SS. | Dead. | |
| " " | 450 " | 0.001 " " | S. | SS. | Dead. |
| " " | 420 " | 1.01 " " | SS. | Dead. | |

2. Tests for strength of antitoxin:

| | Grms. | C.c.toxin. | Antitoxin. | Days. 1st. | 2d. | 3d. | 4th. | 7th. |
|---|---|---|---|---|---|---|---|---|
| Guinea-pig, | 420 | 0.01 | 0.00005 | OK. | OK. | OK. | OK. | OK. |
| " " | 440 | 0.01 | 0.00005 | " | " | " | " | " |
| " " | 510 | 0.01 | Control | SS. | SSS. | Died. | | |
| " " | 540 | 0.01 | Control | S. | SS. | SSS. | Died. | |

The letter S. signifies stiff, SS. increased stiffness, etc.

From these tests we conclude that the serum contains immunizing powers in proportion of 1 : 5,000,000 per body-weight.

The methods of immunization of animals with tetanus antitoxin depend entirely upon circumstances. In cases of penetrating street nail, or any wound in which the presence of tetanus bacilli would be suspected, it would be best to inject at once hypodermatically, with antiseptic precautions, 10 c.c. of the antitoxin, to be followed in a few days with the same amount. The place most convenient for the injection is under the mane or in the region of the shoulder, so as not to interfere with the harness during absorption. Since the period of incubation varies, it is well not to delay after the injury, for after the symptoms are pronounced it requires larger amounts to produce an effect than are practicable in general practice. In case the practitioner has access to the patient at all times, the best rule would be to keep it constantly immunized; in such cases smaller amounts of the antitoxin will suffice. The method most universally adopted is to inject about 3 c.c. of antitoxin of 1:5,000,000 strength into the animal which is to be immunized, and follow this fourteen days later by the same amount, and if the strength of the antitoxin be accurately expressed the animals will be safe from exposure to the disease for about three months. However, when the injuries upon the animal are numerous, and when the cleanliness is neglected, it would be well to repeat the injection again in about two months. We find the best method to treat our animals—which, in the course of immunization to diphtheria and other toxins, and in consequence sufferers from numerous accidental local suppurative lesions—is to inject them once a month with about 10 c.c. of 1 : 5,000,000 antitoxin

The value of this method is shown by the following remarkable statistics collected in the course of a number of years' work in the large antitoxin stables of the H. K. Mulford Company:

In the early part of our experimentation no knowledge indicated to us the necessity of protecting the horses against tetanus, and no precautions were taken to prevent its occurrence. The result was that horse after horse became infected with the disease and died. The local necrosis following the toxic administrations frequently suppurated, and it was impossible to predict what horse might develop tetanus until the initial symptoms appeared. We are at a loss to explain accurately the avenue or origin of the infection, but remembering that the tetanus bacillus is found chiefly in garden-earth, and that garden-earth must make up a large percentage of the dust of hay, and also that this hay-dust is widely distributed in the stable during feeding, falling upon the horses as well as their open wounds, we imagine that the dust was the cause of the infection. Our first attempts at stamping out the disease were, therefore, directed to thoroughly cleansing and disinfecting the stable, bathing the horses with disinfecting solutions, and preventing the distribution of the dust. In spite of the utmost precautions in these directions the death-rate from the dreaded disease at times rose so high as to make us seriously consider abandoning the quarters or temporarily suspending business. The highest death-rate from tetanus alone was in 1898, when it reached 10 per cent. It was after experience such as this that we began to use antitoxin, but the immediate results were most disappointing, because insufficient doses of what was probably a very inferior antitoxin were used. The improvement was most remarkable after we began the systematic immunization of every horse with antitoxin of our own manufacture, since when the death-rate has descended to 1 per cent.

1897.—No precautions taken; death-rate, 8 per cent.

1898.—Poor antitoxin used unsystematically and in insufficient doses; death-rate, 10 per cent.

1899.—Systematic immunization of every horse; death-rate, 1 per cent.

These figures speak eloquently for the advantage of immunization and necessity of sufficient dosage of efficient antitoxin.

It is most difficult to express judgment upon the curative powers of the antitoxin in the absence of accurate figures, for no means are at hand for determining the total number of cases that occur, the number of those that recover spontaneously, or the number that recover under other methods of treatment. In general it must be concluded that the majority of cases of developed tetanus die in spite of the usual therapeutic antitoxin injections, and disappointment is the rule when cures were too sanguinely hoped for.

The occasional absolute futility of injecting 10 c.c. of the antitoxic serum as a therapeutic dose is shown by the fact that we have administered as much as 720 c.c. in one case without any visible beneficial result. In this case the doses were administered as follows: October 19, 1897, A.M., 10 c.c.; P.M., 10 c.c. October 20, 1897, 150 c.c.; October 21, 1897, 250 c.c.; October 22, 1897, 300 c.c. Died. On the other hand, we have seen three horses recover from typical attacks of tetanus after the administration of doses of the antitoxin varying from 150 c.c. administered in daily doses of 25 c.c. to 450 c.c., administered in two large doses.

Of the intracerebral antitoxin injections we can give no information from our experience. They are no longer so highly thought of as formerly. Their use seems to us to be very irrational.

